# Supplementary material for: Love-thy-neighbor: neural networks for tracking and lineage tracing in budding yeast
Source: Bioinform Adv. 2026 Feb 26;6(1):vbag067. doi: 10.1093/bioadv/vbag067 (PMC13169175; doi:10.1093/bioadv/vbag067)
Supplement: vbag067_Supplementary_Data [file vbag067_supplementary_data.docx]

Supplementary Table 1: Performance assessment of TracX and LYN by F1-scores.

| Recording | TracX | LYN-Track |
| --- | --- | --- |
| TTS-YIT-TS1-Sc | 1 | 1 |
| TTS-YIT-TS2-Sc | 1 | 1 |
| TTS-YIT-TS3-Sc | 1 | 0.9972 |
| TTS-YIT-TS4-Sc | 0.9368 | 0.9744 |
| TTS-YIT-TS5-Sc | 0.9891 | 0.9983 |
| TTS-YIT-TS6-Sc | 1 | 1 |
| TTS-YIT-TS7-Sc | 1 | 0.9954 |

Supplementary Table 2: Tracking cells by minimizing the L2 norm with the same features used in LYN-Track.

| Dataset name | Subset | F1-score $\pm$ STD |
| --- | --- | --- |
| SJR6-Sc (validation set) | 5 min | 0.7 $\pm$ 0.2 |
|  | 10 min | 0.6 $\pm$ 0.3 |
|  | 15 min | 0.5 $\pm$ 0.3 |
|  | 20 min | 0.4 $\pm$ 0.3 |
| SJR7-Sc (test set) | 5 min | 0.9 $\pm$ 0.1 |
|  | 10 min | 0.9 $\pm$ 0.2 |
|  | 15 min | 0.8 $\pm$ 0.2 |
|  | 20 min | 0.8 $\pm$ 0.2 |

Supplementary Table 3: LYN-track applied to mammalian cell nuclei timelapse recordings Maška et al. (2023).

| Dataset name | Subset | F1-score $\pm$ STD |
| --- | --- | --- |
| Fluo-N2DH-GOWT | 01 | 0.99 $\pm$ 0.03 |
| Fluo-N2DH-GOWT | 02 | 0.99 $\pm$ 0.02 |
| Fluo-N2DL-HeLa | 01 | 0.4 $\pm$ 0.3 |
| Fluo-N2DL-HeLa | 02 | 0.3 $\pm$ 0.1 |

Supplementary Table 4: Additional features considered but not used for lineage tracing since they did not increase the performance.

| Features related to the growth of the bud |
| --- |
| Speed with which bud’s CoM moves away from candidate mother CoM |
| Features related to the orientation of the bud |
| Bud orientation at the time of budding |
| Standard deviation of the bud orientation |
| Maximum of bud orientation |
| Features related to the position of the bud along the contour of the candidate mother |
| Position angle at time of budding |
| Position angle *N* frames after budding |

Supplementary Table 5: LYN-trace trained and evaluated with only one hidden layer.

| Dataset name | F1-score $\pm$ STD |
| --- | --- |
| SJR* (validation set) | 0.73 $\pm$ 0.02 |
| SJR* (test set) | 0.70 $\pm$ 0.01 |
| TTS-SC7-Sc | 0.837 $\pm$ 0.008 |
